# Correction

**DOI:** 10.1080/2162402X.2025.2517490

**Published:** 2025-06-19

**Article title: Anti-CD47 treatment enhances anti-tumor T-cell immunity and improves immunosuppressive environment in head and neck squamous cell carcinoma**

**Authors**: Lei Wu, Guang-Tao Yu, Wei-Wei Deng, Liang Mao, Lei-Lei Yang, Si-Rui Ma, Lin-Lin Bu, Ashok. B. Kulkarni, Wen-Feng Zhang, Lu Zhang, Zhijun Sun

**Journal**: *OncoImmunology*

**Bibliometrics**: Volume 7, Number 4, pages 1 - 12

DOI: http://dx.doi.org/10.1080/2162402X.2017.1397248

Upon a thorough review of our previous research work, the authors identified some images were incorrect. Specifically, the hematoxylin-eosin (HE) image of Oral Mucosa in Fig. 1A, the immunohistochemical (IHC) image of Foxp3 and CD11b in Fig. 2A and Foxp3 for the CD47 treatment group in Fig.5C were mistakenly used. All authors have confirmed these mistakes do not affect the conclusions of this article. The authors sincerely apologize for these errors.

**Figure 1.**
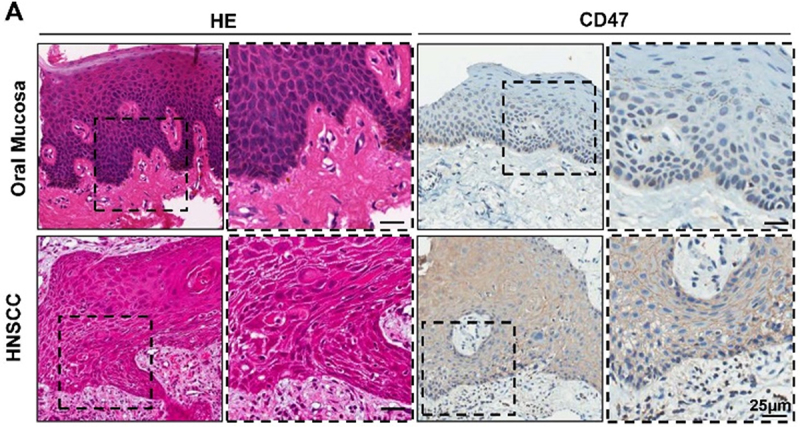
Overexpression of CD47 in human HNSCC confers poor prognosis of HNSCC patients. Representative hematoxylin-eosin, immunohistochemical staining (A) and quantification (B) of CD47 in human head neck squamous cell carcinoma (HNSCC, *n* = 165) as compared with dysplasia (Dys, *n* = 48) and oral mucosa (*n* = 43). Each dot is presented as an independent core. Scale bar, 25μm.

**Figure 2.**
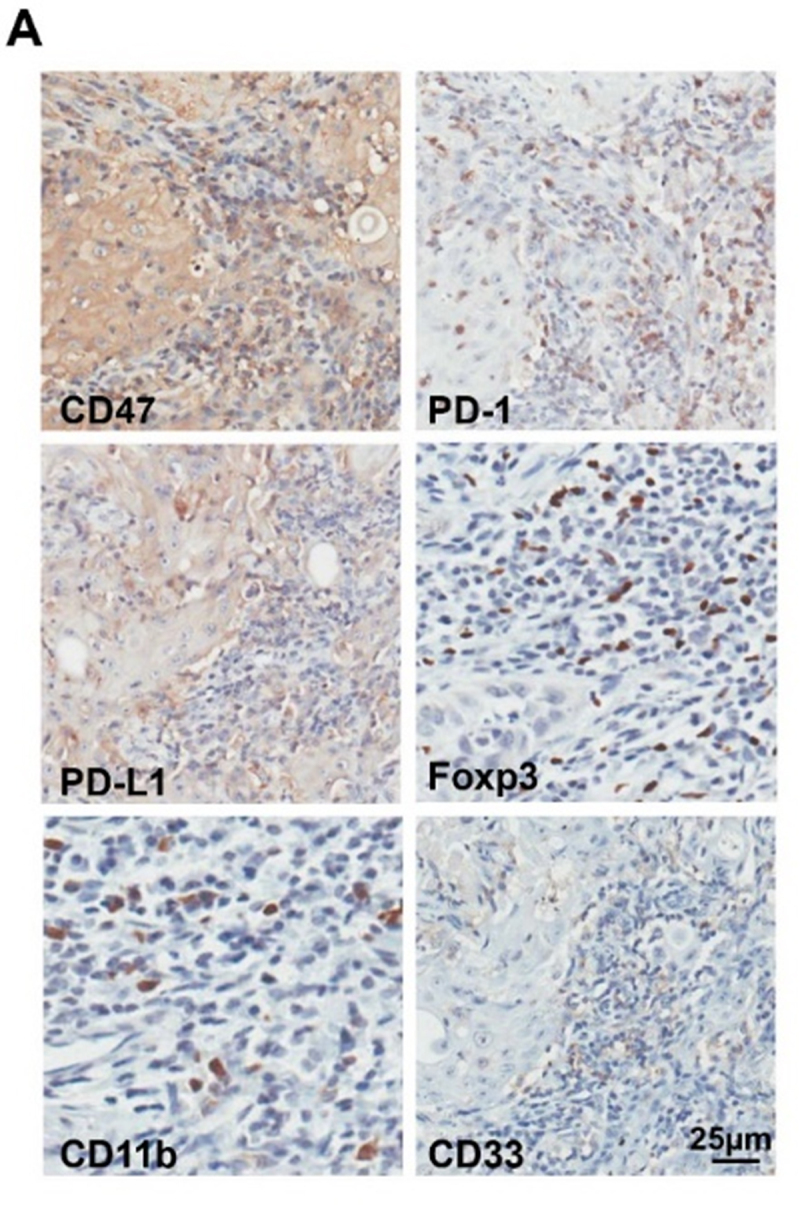
Overexpression of CD47 in human HNSCC is associated with inhibitory checkpoints and suppressive immune cell. (A) Representative immunohistochemical staining of CD47, PD-1, PD-L1, Foxp3, CD11b and CD33 in human HNSCC specimens. Scale bar, 25μm.

**Figure 5.**
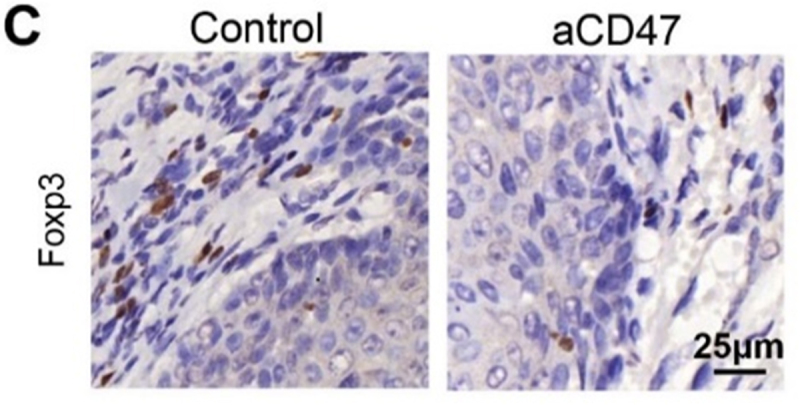
Anti-CD47 treatment significantly attenuated Tregs and MDSCs in Tgfbr1/Pten 2cKO mouse.(C) Immunohistochemical staining of Foxp3 in aCD47 treated mouse HNSCC as compared with isotype IgG treated counterpart. Scale bar, 25μm.

**Corrected Fig. 1A**

**Corrected Fig. 2A**

**Corrected Fig. 5C**

